# Sex-specific medication trajectories in older adults newly diagnosed with diabetes

**DOI:** 10.1016/j.rcsop.2023.100294

**Published:** 2023-06-15

**Authors:** Miceline Mésidor, Denis Talbot, Marc Simard, Claudia Blais, Véronique Boiteau, Caroline Sirois

**Affiliations:** a Département de médecine sociale et préventive, Université Laval, Pavillon Ferdinand-Vandry 1050, Avenue de la Médecine, Québec G1V 0A6, Canada; b Centre de Recherche du CHU de Québec – Université Laval, 2400 Av. D'Estimauville, Québec G1E 6W2, Canada; c Faculté de Pharmacie, Université Laval, Pavillon Ferdinand-Vandry, 1050 Av. de la Médecine, Québec G1V 0A6, Canada; d Institut National de Santé Publique du Québec, 945, av Wolfe, Québec G1V 5B3, Canada

**Keywords:** Diabetes, Medication use, Older adults, Sex differences, Latent class models, Quebec

## Abstract

**Background:**

People with diabetes tend to use many medications to treat diabetes and comorbidities. Nevertheless, the evolution of polypharmacy in newly diagnosed males and females has been little studied.

**Objective:**

The objective of this paper was to identify and describe medication trajectories in incident diabetes cases according to sex.

**Methods:**

Data were obtained from the Quebec Integrated Chronic Disease Surveillance System. We built a population-based cohort of community-dwelling individuals aged >65 years diagnosed with diabetes in 2014 who were alive and covered with the public drug plan until March 31, 2019. Latent class models were used to identify medication trajectory groups in males and females separately.

**Results:**

Of the 10,363 included individuals, 51.4% were males. Females were older and more likely to have more medication claims than males. Four trajectory groups were identified for males and five for females. Most trajectories showed sustained and stable number of medications over time. For each sex, only one of the trajectory groups included a mean annual number of medications lesser than five. Slight increasing trends of medication use were detected in the trajectories composed of very high users, which included older, more comorbid individuals frequently exposed to potentially inappropriate medications.

**Conclusions:**

Most males and females with incident diabetes had a high burden of medication following the year of diagnosis and were classified in a group of sustained medication use over time. The largest increase in medication was among those who had higher level of polypharmacy of questionable quality at baseline, raising concerns about the innocuity of such medication trajectories.

## 1 Introduction

Diabetes is one of the most common chronic diseases among older adults. In the province of Quebec, Canada, 21.2% of individuals aged 65 to 74 years, and 28.1% of those aged 75 years and older had a diagnosis of diabetes in 2019.^1^ Diabetes is also associated with many comorbidities such as coronary disease, stroke, peripheral arterial disease, and renal impairment.^2^ Over 97% of older persons with diabetes have at least one comorbidity and >40% have at least four.^3^ Understandably, the total health care costs related to diabetes are high; in 2022, the estimates were 15.4 billion dollars in Canada.^4^ As the population ages, these costs will increase over time. Optimal treatments that may prevent complications are thus essential to reduce the clinical and financial burden of this disease.

Medication plays an important role in treating and preventing diseases. Older adults with diabetes are particularly prone to use many medications, both to treat diabetes and other concomitant conditions. Indeed, the average number of medications used by older adults with diabetes in Quebec in 2014 was 12.^5^ As a result, most individuals with diabetes are exposed to polypharmacy, which is most commonly defined as the concomitant use of at least five medications.^6^^,^^7^ However, polypharmacy has been associated with adverse outcomes, such as increased falls, hospitalizations, and mortality.^8^ The adverse effects of polypharmacy are not only related to the number of medications but also to their appropriateness.^9^^,^^10^ In fact, a higher number of medications increases the risk of using potentially inappropriate medications,^11^ that is medications for which benefits are lower than their risks or for which other safer alternatives exist.^12^ Many lists can be used to identify potentially inappropriate medications, such as the Beers' criteria.^13^

Longitudinal studies can help to capture heterogeneity in medication use and to assess changes in individuals' medication use over time in order to better target high medications users, and those at higher risk of potentially inappropriate medication use. Latent class growth analysis (LCGA) is a special case of latent class growth mixture models that identify groups of individuals with similar patterns.^14^ This method has been used in the diabetes literature to study medication adherence patterns,^15^ depression or anxiety symptoms,^16^, ^17^, ^18^, ^19^, ^20^ patterns or health outcomes trajectories.^21^, ^22^, ^23^ However, to our knowledge, no study focused on medication patterns over time among older adults with incident diabetes. Exploring such patterns would help determine if, and to what extent, the burden of treating diabetes and comorbidities increases following diagnosis, and which patients are the most at risk of potentially inappropriate polypharmacy, which would help optimize pharmacotherapy.

Sex-differences exist in the prevalence of polypharmacy among older adults. Several studies have shown that females are generally more exposed to polypharmacy than males.^24^^,^^25^ However, according to a recent study in Canada, although the prevalence estimates of multimorbidity and polypharmacy were greater in females than males in 2003 and 2016, the absolute increase was greater for males over time,^26^ which narrowed the gap in 2016. To our knowledge, such a comparison of usage patterns between males and females has not been conducted in people newly diagnosed with diabetes.

The objectives of this paper were twofold: i) to identify trajectories of medications use in the four years following the diagnosis of diabetes, separately for males and females; ii) to describe the baseline characteristics associated with each trajectory, including medication classes and potentially inappropriate medications claimed in the year after the diagnosis.

## 2 Methods

The Quebec Integrated Chronic Disease Surveillance System (QICDSS) is a system developed and managed by the *Institut national de santé publique du Québec* (INSPQ) for chronic disease surveillance.^27^ The QICDSS includes five health administrative databases. Data on socio-demographic characteristics, eligibility and admissibility to the Quebec public health and drug insurance are included in the health insurance registry. The physician claims and hospitalization databases contain data on health services and hospitalizations, respectively. Data on death are recorded in the vital statistics death registry. Prescription medications information (e.g., name of the medications) is contained in the pharmaceutical services database. Medication data from the QICDSS cover >90% of community-dwelling adults aged 65 and older and these health administrative databases have been previously validated.^28^, ^29^, ^30^

The study population includes individuals aged 66 years or older as of April 1st, 2015, with a diagnosis of diabetes between April 1st, 2014, and March 31st, 2015. Individuals were followed until March 31st, 2019. We included participants who were entirely covered by the universal Public Prescription Drug Insurance Plan between April 1st, 2015, and March 31st, 2019, to better represent medication use. Individuals who were transferred to long-term care facilities were excluded because data on medications were not available. Finally, only individuals with at least three years of follow-up were included.

**Fig. 1:**
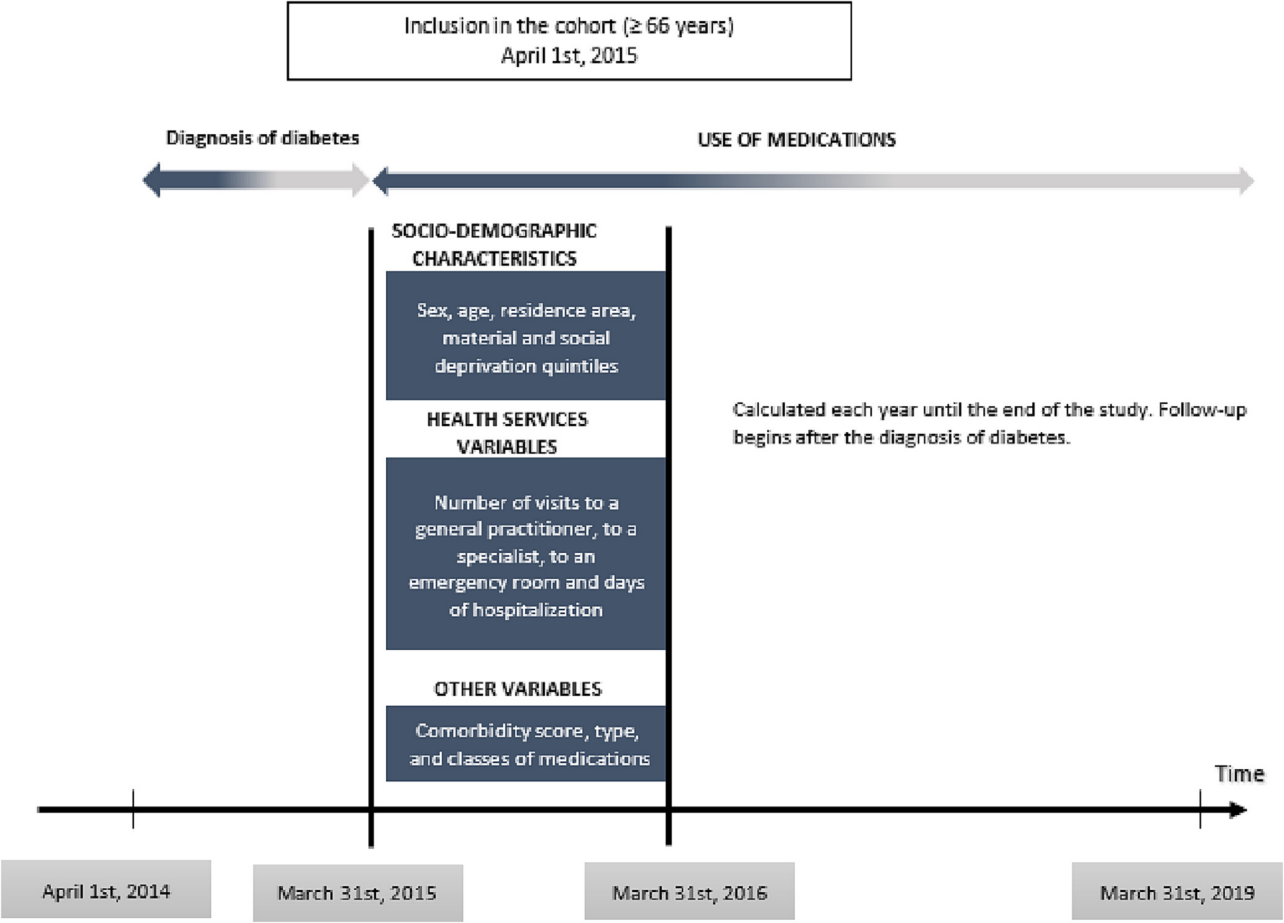
Creation of the cohort of older adults newly diagnosed with diabetes in the province of Quebec, Canada.

To identify individuals newly diagnosed with diabetes, we used a validated case definition (sensitivity = 94.6% and positive predictive value = 87.9%) routinely used in surveillance in Canada.^5^^,^^31^ Subjects with two physicians claims with a diagnosis of diabetes (International Classification of Diseases - 9th version, ICD-9: 250, ICD-10: E10-E14) within two years or one hospitalization with such a diagnosis code were considered as having diabetes.^27^ The date of diagnosis was the earliest of the second date of the physician claims, or the date of hospital discharge. The creation of the cohort is illustrated in Fig. 1.

The common denomination was used to identify the medications claimed by each subject.^32^ It corresponds to the 5th level of the Anatomical Therapeutical Chemical (ATC) classification. We considered subjects to be using a specific medication during a year if there was at least one claim for that medication during that year. For each fiscal year, we counted the total number of different medications claimed by each individual, regardless of the class of the medications.

We considered 11 baseline characteristics that were used as covariates. They included demographic variables [sex (male/female), age (in years), residence area (urban [≥10,000 inhabitants]/rural), material and social deprivation quintiles^33^], variables of health service utilization (number of visits to a general practitioner, a specialist, an emergency room and number of days of hospitalization), comorbidity score^34^ and medication variables (classes of medications, potentially inappropriate medications). The material and social deprivation quintiles,^33^ validated ecological proxies to socioeconomic status, were constructed from census data on the Quebec population, with quintile 1 comprising the most privileged and quintile 5 the most deprived subjects. The material deprivation quintile includes information on education, income, and employment status while the social deprivation quintile considers people who live alone in their homes, their marital status, and single-parent families. Comorbidities were assessed based on the combined comorbidity index of Charlson and Elixhauser (2018) that contains a list of 32 conditions with weights of comorbidities, using Charlson weights, ranging from 0 to 5.^34^ This index was validated in the Quebec population and provided high discrimination results in predicting mortality.^34^ We categorized the medication classes that were claimed following the year of diagnosis with the use of the 3rd level of the ATC classification. We also identified potentially inappropriate medications claimed in this period using the 2015 Beers criteria,^13^ which were previously adapted to our datasets (Table S1).^11^^,^^35^^,^^36^

We first described the characteristics of the included individuals using means and standard deviations for the continuous variables and proportions for the categorical variables. LCGA was used to identify groups of medication trajectories. Models with 1–10 trajectories were considered and the model with the lowest Bayesian information criterion (BIC) among those with at least 5% of individuals per group was selected. Once the number of groups was selected, the shapes of the trajectories were determined by modeling cubic polynomials and excluding higher order polynomial terms not significant at the 5% level. Participants were allocated to the trajectory group for which their posterior probability was highest. As recommended by several authors,^22^^,^^37^ we assessed the validity of trajectory groups using different procedures. We first computed three model adequacy criteria: the average posterior probability (values >70% indicate adequate classification), the odds of correct classification (values >5 suggest high accuracy in the assignment of trajectories) and the relative entropy (values above 0.8 imply less uncertainty in the classification). If any of the criteria were below the threshold, a model with fewer groups was considered. Then, we applied a method based on bootstrap to assess the uncertainty in the number of groups.^38^ We considered 100 bootstraps samples. All analyses were stratified by sex. Finally, for the second objective, we used descriptive statistics to present the characteristics of individuals in each trajectory. Analysis of variance and chi-square tests were used to assess statistical differences between demographic, health utilization and medication variables and identified trajectories with a level of significance α = 5%.

As a sensitivity analysis, we identified medication trajectories among participants newly diagnosed with diabetes and a comorbidity score of zero. This analysis aimed to better isolate the impact of the diagnosis of diabetes on medication use among individuals who do not present major comorbidities and therefore are not expected to use many medications at baseline.

Analyses were performed using SAS 9.4 software (SAS Institute, Cary, NC) and R.4.1.0 software (© 2021 The R Foundation for Statistical Computing, Austria, package *FlexMix*).^39^

The process for creating and for accessing the QICDSS data meet stringent standards of security and privacy. The use of these data was approved by the Commission d'accès à l'information du Québec and the government bodies (Régie de l'assurance de la maladie du Québec, Ministère de la Santé et des Services sociaux). This study meets all QICDSS data requirements for surveillance purposes and consent was not required for the participants.

## 3 Results

Table 1 presents the baseline characteristics of the 10,363 included individuals. Of them, 5330 (51.4%) were males. Participants were on average 74.2 years (standard deviation: SD = 6.3), had a mean comorbidity score of 2.7 (SD = 3.4) and a mean annual number of medications of 10.4 (SD = 5.9). Descriptively, females were older, more likely to be from an urban area, to be socially deprived, to claim more medications, and to have more encounters with a generalist in 2015 than males.

**Table 1:** Characteristics of participants 66 years and older with a new diagnosis of diabetes, by sex, in the province of Quebec, Canada, 2015.

Variables	Total (*n* = 10,363)	Males (*n* = 5330)	Females (*n* = 5033)
Age, mean (SD)^1^	74.2 (6.3)	73.6 (5.8)	74.9 (6.7)
Residence area^2^, n (%)			
Urban	8032 (77.6)	4016 (75.3)	4016 (79.8)
Rural	2332 (22.4)	1311 (24.6)	1011 (20.1)
Material deprivation quintile^2^, n (%)			
Quintile 1 (most privileged)	1445 (15.4)	776 (15.8)	669 (15.0)
Quintile 2	1593 (17.0)	852 (17.3)	741 (16.6)
Quintile 3	1821 (19.4)	944 (19.2)	877 (19.6)
Quintile 4	2134 (22.8)	1098 (22.4)	1036 (23.2)
Quintile 5 (most deprived)	2384 (25.4)	1242 (25.3)	1142 (25.6)
Social deprivation quintile^2^, n (%)			
Quintile 1 (most privileged)	1677 (17.9)	943 (19.2)	734 (16.4)
Quintile 2	1844 (19.7)	1040 (21.2)	804 (18.0)
Quintile 3	1895 (20.2)	1040 (21.2)	855 (19.1)
Quintile 4	1942 (20.7)	938 (19.1)	1004 (22.5)
Quintile 5 (most deprived)	2019 (21.5)	951 (19.4)	1068 (23.9)
Comorbidity score, mean (SD)^1^	2.7 (3.4)	2.8 (3.5)	2.6 (3.3)
Number of medications used, mean (SD)^1^	10.4 (5.9)	9.7 (5.7)	11.2 (6.1)
Number of visits to a generalist, mean (SD)^1^	3.4 (3.0)	3.2 (2.8)	3.6 (3.2)
Number of visits to a specialist, mean (SD)^1^	5.3 (7.8)	5.4 (8.1)	5.1 (7.4)
Number of emergency visits, mean (SD)^1^	0.7 (1.4)	0.6 (1.4)	0.7 (1.3)
At least one emergency visits, n (%)	3371 (32.5)	1682 (31.6)	1689 (33.6)
Length (in days) of hospitalization, mean (SD)^1^	2.0 (7.9)	2.0 (8.3)	1.9 (7.5)
Hospitalized at least one day, n (%)	1587 (15.3)	828 (15.5)	759 (15.1)

1 SD: Standard deviation.

2 Sum of counts for residence area, material and social deprivation quintiles does not equal the total number of subjects due to missing values.

**Fig. 2:**
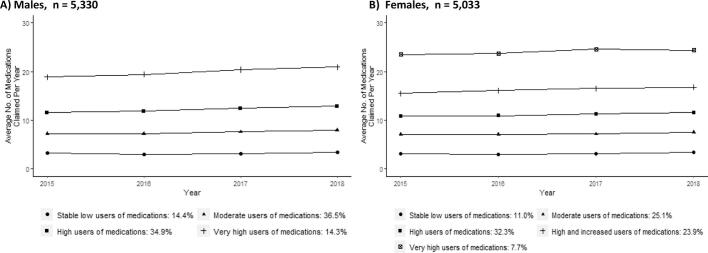
Trajectories of medications used among individuals with a new diagnosis of diabetes, by sex, in Quebec, Canada, *n* = 10,363. *Legend.* The year refers to the fiscal year. For example, medications claimed between April 1st, 2015, and March 31st, 2016, were considered as the number used for 2015.

In the male subpopulation, the BIC suggested four groups of medication trajectories with quadratic shapes (Fig. 2 A, Table S2). All trajectories were characterized by sustained, stable number of medications claimed over the years, apart from the category that used the largest number of medications, whose claims tended to increase. >85% of the male subpopulation was included in trajectories with polypharmacy. Specifically, the largest group, identified as “moderate users of medications” (36.5%, 1946/5330), had an average of 7.1 different medications in 2015. Approximately 35% of individuals pertained to the “high users of medications” (34.9%, 1858/5330) group, that claimed an average of 11.5 different medications. “Stable low users of medications” (14.4%, 766/5330) consistently had low claims of medications (mean = 3.2). In contrast, 14.3% (760/5330) were included in the “very high users of medications” group, that claimed an average of 18.7 different medications and gradually increased the number of medications.

There were significant differences in baseline characteristics between the four groups. We observed a constant progression in the age of the individuals, their comorbidity score and their use of health services according to the increase in the number of medications in the trajectory groups (Table 2). Similarly, the proportion of users of the different classes of medications at baseline increased steadily from the trajectory of “stable low users of medications” to the one of “very high users of medications” (Fig. S1, A). Medication classes used in diabetes and cardiovascular diseases (lipid modifying agents, agents acting on the renin-angiotensin system, beta blocking agents and calcium channel blockers) were frequently claimed across all groups, with lipid modifying agents being the most frequent category. All in all, the relative importance of each medication classes used was very similar through the trajectories, except that in the “high users of medications” and the “very high users of medications” groups, a higher proportion of individuals claimed analgesics and drugs for acid related disorders. The pattern of use of potentially inappropriate medication was somewhat less related to the gradient of medication trajectories, but “high users of medications”, and especially “very high users of medications” were more likely to claim them. >40% of “very high users of medications” had a claim for potentially inappropriate medications of the central nervous system (Fig. S2).

**Table 2:** Characteristics of older males newly diagnosed with diabetes by trajectory groups in Quebec, Canada, *n* = 5330, 2015.

	Stable low users of medications (*n* = 766)	Moderate users of medications (*n* = 1946)	High users of medications (*n* = 1858)	Very high users of medications (*n* = 760)	*p*-value
Age, mean (SD)^1^	72.3 (5.3)	73.1 (5.5)	74.1 (6.0)	75.0 (6.3)	<0.001
Residence area, n (%)					
Urban	594 (77.5)	1508 (77.5)	1362 (73.4)	552 (72.7)	0.004
Rural	172 (22.5)	438 (22.5)	493 (26.6)	208 (27.4)	
Material deprivation quintile^2^, n (%)					0.289
Quintile 1 (privileged)	118 (16.3)	312 (17.4)	249 (14.5)	97 (14.3)	
Quintile 2	115 (15.9)	326 (18.1)	291 (17.0)	120 (17.7)	
Quintile 3	148 (20.4)	337 (18.7)	339 (19.8)	120 (17.7)	
Quintile 4	166 (22.9)	384 (21.4)	378 (22.1)	170 (25.1)	
Quintile 5 (deprived)	177 (24.4)	439 (24.4)	455 (26.6)	171 (25.2)	
Social deprivation quintile^2^, n (%)					0.112
Quintile 1 (privileged)	162 (22.4)	354 (19.7)	309 (18.1)	118 (17.4)	
Quintile 2	152 (21.0)	391 (21.7)	355 (20.7)	142 (20.9)	
Quintile 3	147 (20.3)	394 (21.9)	365 (21.3)	134 (19.8)	
Quintile 4	129 (17.8)	349 (19.4)	325 (19.0)	135 (19.9)	
Quintile 5 (deprived)	134 (18.5)	310 (17.2)	358 (20.9)	149 (22.0)	
Comorbidity score, mean (SD)^1^	1.5 (2.5)	2.0 (2.7)	3.2 (3.4)	5.6 (4.3)	<0.001
Number of medications used, mean (SD)^1^	3.2 (2.1)	7.1 (2.2)	11.5 (3.2)	18.7 (6.0)	<0.001
Number of visits to a generalist, mean (SD)^1^	2.0 (1.9)	2.7 (2.2)	3.5 (2.8)	4.7 (3.8)	<0.001
Number of visits to a specialist, mean (SD)^1^	2.6 (3.6)	3.7 (4.9)	6.3 (7.2)	10.8 (14.6)	<0.001
Number of emergency visits, mean (SD)^1^	0.2 (0.7)	0.3 (0.8)	0.7 (1.2)	1.7 (2.5)	<0.001
At least one emergency visits, n (%)	128 (16.7)	425 (21.8)	672 (36.2)	457 (60.1)	<0.001
Length (in days) of hospitalization, mean (SD)^1^	0.5 (3.3)	0.7 (5.7)	2.1 (7.5)	6.5 (14.8)	<0.001
Hospitalized at least one day, n (%)	43 (5.6)	168 (8.6)	331 (17.8)	286 (37.6)	

1 SD: Standard deviation.

2 Sum of counts for residence area, material and social deprivation quintiles does not equal the total number of subjects due to missing values.

In the female subpopulation, the optimal model suggested by the BIC was five groups with quadratic shapes (Fig. 2 B,Table S2). Similar to the male subpopulation, all trajectories showed a sustained and stable number of medications except two which presented a trend toward increased use, but not as large as that identified in the male subpopulation. Nine out of ten (89%) women were included in trajectories with polypharmacy. The largest group “high users of medications” (32.3%, 1625/5033) claimed constant high number of medications over time (mean = 10.8 in 2015) (Fig. 2, B).

The differences in baseline characteristics between the groups followed the same patterns as observed in men; the more medications the trajectories include, the older the individuals, the higher their comorbidity score and the greater the use of health services (Table 3). However, the distribution of the classes of medications was more heterogeneous between the groups in female than male, and the proportions of users did not increase steadily across trajectories as was observed in the male subgroup (Fig. S1, B). For example, while lipid modifying agents, agents acting on the renin-angiotensin system and drug used in diabetes were again frequent medication classes, there was no evident increase in the proportion of users beyond the trajectory of “high users of medications”. The most frequent class of medications claimed in the two groups of very high use and high and increased use of medications were drugs for acid related disorders and analgesics, classes that were also identified among men. Trends for the use of potentially inappropriate medications were more present in females than males, with the proportion of users increasing with the increase in medication trajectories. The pattern was most prominent with central nervous system medications, where >50% of “very high users of medications” and “high and increased users of medications” had a claim for a medication in this category (Fig. S2).

**Table 3:** Characteristics of older females newly diagnosed with diabetes by trajectory groups, Quebec, Canada, *n* = 5033, 2015.

	Stable low users of medications (*n* = 552)	Moderate users of medications (*n* = 1262)	High users of medications (*n* = 1625)	High and increased users of medications (*n* = 1205)	Very high users of medications (*n* = 389)	p-value
Age, mean (SD)^1^	72.81 (5.92)	73.81 (6.21)	75.33 (6.84)	76.22 (6.76)	75.90 (7.06)	<0.001
Residence area, n (%)						0.001
Urban	454 (82.2)	1052 (83.4)	1276 (78.6)	940 (78.2)	296 (76.1)	
Rural	98 (17.8)	210 (16.6)	348 (21.4)	262 (21.8)	93 (23.9)	
Material deprivation quintile^2^, n (%)						0.002
Quintile 1 (privileged)	92 (17.6)	183 (16.0)	204 (14.3)	157 (15.2)	33 (9.7)	
Quintile 2	99 (19.0)	171 (14.9)	247 (17.3)	168 (16.2)	56 (16.5)	
Quintile 3	98 (18.8)	254 (22.2)	266 (18.6)	178 (17.2)	81 (23.9)	
Quintile 4	96 (18.4)	261 (22.8)	358 (25.1)	252 (24.3)	69 (20.4)	
Quintile 5 (deprived)	136 (26.1)	275 (24.0)	351 (24.6)	280 (27.1)	100 (29.5)	
Social deprivation quintile^2^, n (%)						0.475
Quintile 1 (privileged)	102 (19.5)	197 (17.2)	227 (15.9)	153 (14.8)	55 (16.2)	
Quintile 2	100 (19.2)	188 (16.4)	260 (18.2)	188 (18.2)	68 (20.1)	
Quintile 3	96 (18.4)	238 (20.8)	261 (18.3)	206 (19.9)	54 (15.9)	
Quintile 4	110 (21.1)	247 (21.6)	332 (23.3)	240 (23.2)	75 (22.1)	
Quintile 5 (deprived)	113 (21.6)	274 (24.0)	346 (24.2)	248 (24.0)	87 (25.7)	
Comorbidity score, mean (SD)^1^	0.93 (1.86)	1.53 (2.44)	2.46 (3.10)	3.66 (3.49)	5.42 (4.01)	<0.001
Number of medications used, mean (SD)^1^	3.17 (2.23)	7.02 (2.08)	10.84 (2.65)	15.60 (3.69)	23.48 (5.65)	<0.001
Number of visits to a generalist, mean (SD)^1^	2.21 (1.94)	2.77 (2.10)	3.37 (2.65)	4.58 (3.65)	6.48 (5.37)	<0.001
Number of visits to a specialist, mean (SD)^1^	2.43 (3.53)	3.13 (4.03)	4.80 (5.54)	7.15 (10.65)	10.53 (9.70)	<0.001
Number of emergency visits, mean (SD)^1^	0.21 (0.61)	0.30 (0.77)	0.56 (1.09)	0.99 (1.48)	2.07 (2.51)	<0.001
At least one emergency visits, n (%)	77 (13.9)	245 (19.4)	519 (31.9)	587 (48.7)	261 (67.1)	<0.001
Length (in days) of hospitalization, mean (SD)^1^	0.32 (2.10)	0.54 (2.64)	1.41 (6.62)	3.10 (9.62)	7.50 (13.40)	<0.001
Hospitalized at least one day, n (%)	22 (4.0)	89 (7.1)	201 (12.4)	263 (21.8)	184 (47.3)	<0.001

1 SD: Standard deviation.

2 Sum of counts for material and social deprivation quintiles does not equal the total number of subjects due to missing values.

The three model adequacy criteria were above the recommended threshold suggesting adequate criteria for both subpopulations (Table S3). Of the 100 bootstraps samples, among the male subpopulation, 59 selected four groups and 35 six groups (Table S4), in which two groups contained no individuals and the trajectories obtained were the same as those of the four-group model. Among the female subpopulation, 86 of the 100 bootstrap samples selected five groups and seven selected four and six groups, respectively. These results support that the number of groups found is adequate. In addition, the results of the sensitivity analysis were similar to those of the main analysis; the trajectory groups of individuals with a comorbidity score of zero were predominantly like those in the general population (Supplementary material B, Fig. S3).

## 4 Discussion

In this population of individuals newly diagnosed with diabetes, we found that for both male and female, <15% had low medication claims or no polypharmacy. Trajectories of medication claims were mostly stable over time, but the increase of medications claims was faster among people who had claims for more medications at baseline. No trajectory suggested a decrease in medication, which could have been the result of deprescribing.

For most trajectories, there was no obvious increase in the number of medications claimed in the year following the diagnosis of diabetes. The fact that the number of medications was already very high immediately after the diagnosis of diabetes may partly explain this observation, as many cardioprotective medications, for example, were already used. Interestingly, the diagnosis of diabetes did not seem to cause prescribing cascades or lead to other conditions that would increase the use of medications, which is reassuring from a clinical and public health perspective. This also held true for the subpopulation with a comorbidity score of 0. However, the fact that the trajectories with the highest medication use were the ones where increasing trends were seen warrants caution, as these individuals already appeared the most vulnerable to the adverse effects of polypharmacy because of their baseline characteristics and the fact that they were more often exposed to potentially inappropriate medications. Other studies have described trajectories in other populations and found that more than half of included participants were in the trajectory of high level medications use over time.^9^^,^^40^ Although the study population was different than ours and does not include only newly diagnosed individuals, their results also show that high use of medications persists over time and is not only related to people with diabetes.

Differences in medication claims according to sex were observed. We found that females tended to have higher levels of medication claims than males, which is concordant with other studies.^26^ Differences in medication use at baseline was also significant between trajectories and sex. While medication classes pertaining to the prevention/treatment of cardiovascular comorbidities were consistently prominent in all male-specific trajectories, medication classes used at baseline were more heterogenous in female-specific trajectories. The diversity of medication classes can be explained by the use of sex-specific medications, such as calcium and vitamin D use in women. However, some gender disparities may also be present, as the use of cardioprotective treatments have been shown to be used less frequently among women than men.^41^ Of note, the use of analgesics was frequent for both men and women in trajectories with high medication use, suggesting that pain may represent a significant issue among those individuals. However, our analysis does not permit to evaluate if this condition was acute or chronic.

The use of potentially inappropriate medications was mostly problematic in trajectories with a high number of medications. Medications acting on the central nervous system was particularly significant, which is concordant with the results of a population-based study conducted in Taiwan from 2013 to 2015 in older adults with chronic diseases.^42^ Beers criteria recommend that tricyclic antidepressants, paroxetine and benzodiazepines, for example, should be avoided in older adults because of their associated increased risk of anticholinergic effects and cognitive impairment, among others.^13^ Furthermore, using a combination of three or more certain central nervous system medications can increase the risk of falls or fracture.^13^ Again, since the individuals in the trajectory groups with many medications presented characteristics that made them vulnerable, the associated risks might be even greater for this population frequently exposed to these medications.

In this article, we included the entire population that met the inclusion criteria, and the public drug plan covers a wide range of medications. Hence, we are able to describe a very accurate profile of medications claimed. Limitations of this study are related to the nature of the data. Because the data were extracted from health administrative databases, we do not have access to a clinical definition of diabetes. Therefore, we used a validated definition of diabetes to identify diabetes cases. The study population may include some false positives. We do not have the indication for the medications, or the clinical information needed to assess the appropriateness of the treatment. Then, we considered medication claims as use of medications, however, individuals may not use all medications. We also did not look at concurrent medication use. Hence, we have a picture over a whole year and medications taken over a short period of time or for an acute condition will have the same weight as medications taken chronically. Nevertheless, this is the most used way to study polypharmacy or potentially inappropriate medications because it allows an idea of the diversity and complexity of the medications that were used in the year.^11^^,^^43^^,^^44^ We also excluded the sickest people, that is, those who died and those who were admitted to long-term care. However, their consumption might have been different, since end-of-life care is associated with an increase in care, thus their inclusion would likely have generated heterogeneity. Data for non-prescription medications or natural medicines are not available; thus, our results likely underestimate the total number of medications received by older adults. Finally, because our results are based on data from a single province in Canada, they may not generalize to other countries. In particular, medication trajectories may be different in low- and middle-income countries or in jurisdictions where older adults do not benefit from public drug insurance.

## 5 Conclusion

In conclusion, both males and females newly diagnosed with diabetes have a significant baseline medication burden. In this first study to describe medication patterns in newly diagnosed individuals with diabetes, we observed that most individuals have sustained medication use over time and the increase in number of medications is more important among heavy users, which strengthens the importance of considering the longitudinal aspect of polypharmacy. Physicians must be aware that any additional medications will increase polypharmacy; therefore, they should regularly assess the appropriateness of all medications and deprescribe when possible.

## Funding

This work was supported by a 10.13039/100005622Collaborative Health Research Projects grant from the 10.13039/100005622Canadian Institute of Health Research and the 10.13039/501100000038Natural Sciences and Engineering Research Council of Canada (grant number: CPG-170621). Miceline Mésidor is supported by a postdoctoral fellowship from the Fonds de recherche du Québec – Santé (FRQS). Caroline Sirois and Denis Talbot are holders of a Junior 2 salary awards from the FRQS. Marc Simard is supported by a PhD fellowship from the FRQS.

## Authors contributions

MM, CS and DT developed the original idea and designed the analytical plan. MM conducted the literature review, completed the statistical analyses, and drafted the manuscript. MS, VB, and CB were involved in the creation of the cohort study. All authors critically reviewed and approved the manuscript.

## Declaration of Competing Interest

The authors declare that they have no known competing financial interests or personal relationships that could have appeared to influence the work reported in this paper.

## Data Availability

Data used for the analysis are the propriety of the Ministère de la Santé et des Services sociaux du Québec and the Régie de l'assurance maladie du Québec. Access to these data is limited to authorized personnel of the Chronic Disease and Injury Surveillance Unit.

## Appendix A Supplementary data

Supplementary material Image 1
