# Supplementary material for: Sex-specific medication trajectories in older adults newly diagnosed with diabetes
Source: Explor Res Clin Soc Pharm. 2023 Jun 15;11:100294. doi: 10.1016/j.rcsop.2023.100294 (PMC10319302; doi:10.1016/j.rcsop.2023.100294)
Supplement: Supplementary file 1 — Supplementary material [file mmc1.docx]

**Supplementary material A.**

Table S1. List of potentially inappropriate medications included in the study, based on the Beers criteria, 2015

| **Classes of medications** | **Name of medications** | **Common denomination codes** |
| --- | --- | --- |
| **Anticholinergics** | | |
| ***Antihistamines*** | Brompheniramine | 1131, 46058 |
|  | Chlorpheniramine | 1885, 45575, 46107, 46364, 46730 |
|  | Clemastine | 39068 |
|  | Cyproheptadine | 2379 |
|  | Dexbrompheniramine | 46257 |
|  | Dexchlorpheniramine | 2639 |
|  | Dimenhydrinate | 2938, 46212 |
|  | Diphenhydramine | 3107, 46381 (only products for oral use) |
|  | Doxylamine | 46045, 46140, 47131 |
|  | Hydroxyzine | 4706 |
|  | Meclizine | 5512, 46079 |
|  | Promethazine | 8177, 34336, 46439 |
|  | Triprolidine | 9945 |
| ***Antiparkinsonian agents*** | Benztropine | 34323 (only products for oral use) |
|  | Trihexyphenidyl | 9828 |
| ***Antispasmodics*** | Atropine | 689 (only products for oral use), 10881, 18829, 45466, 46721 |
|  | Belladonna alkaloids | 46118, 46307 |
|  | Clidinium-chlordiazepoxide | 46166 |
|  | Dicyclomine | 2756, 46685 |
|  | Hyoscyamine | 46205 |
|  | Propantheline | 8203 |
|  | Scopolamine | 8814, 8827, 34687, 46109, 46827 |
| ***Antithrombotics*** | Dipyridamole | 3094 (only products for oral use), 46077 |
|  | Ticlopidine | 45617 |
| **Cardiovascular** | | |
| ***Central alpha agonists*** | Clonidine | 10751 |
|  | Guanfacine | 47979 |
|  | Methyldopa | 6136 |
| ***Alpha-blockers*** | Doxazosin | 45625 |
|  | Prazosin | 37742 |
|  | Terazosin | 45520 |
| ***Other*** | Disopyramide | 37911, 38080 |
|  | Dronedarone | 47804 |
|  | Nifedipine | 42708 (only products for oral use), 46388 |
| **Central nervous system** | | |
| ***Antidepressants*** | Amitriptyline | 429, 442, 46011 |
|  | Amoxapine | 43696 |
|  | Clomipramine | 14781 |
|  | Desipramine | 2522 |
|  | Imipramine | 4784 |
|  | Nortriptyline | 6578 |
|  | Paroxetine | 47061 |
|  | Protriptyline | 8294 |
|  | Trimipramine | 9906 |
| ***Barbiturics*** | Amobarbital | 468, 481 |
|  | Butabarbital | 1157, 1170 |
|  | Butalbital | 46012, 46013 |
|  | Mephobarbital | 5642 |
|  | Pentobarbital | 7124 |
|  | Phenobarbital | 7345, 7358, 17906, 19128, 46172, 46338, 46558, 46685 |
|  | Secobarbital | 8853 |
| ***Benzodiazepines*** | Alprazolam | 43501 |
|  | Bromazepam | 43488 |
|  | Lorazepam | 37950, 46440 |
|  | Oxazepam | 6786 |
|  | Temazepam | 41590 |
|  | Triazolam | 39029 |
|  | Clorazepate | 14768 |
|  | Chlordiazepoxide | 1807, 46166 |
|  | Clonazepam | 37872 |
|  | Diazepam | 2717, 46161 |
|  | Flurazepam | 4095, 46818 |
|  | Meprobamate | 5681 |
|  | Acetylsalicylic acid | 46368 |
| ***Z-drugs*** | Zolpidem | 47912 |
|  | Zaleplon | 46668 |
|  | Zopiclone | 46047 |
| **Endocrine** | | |
|  | Thyroid extracts | 9659, 43059 |
|  | Estrogens | 43072, 45582 (only products for oral use), 45583, 47395 |
|  | Megestrol | 38483 |
|  | Chlorpropamide | 1937 |
|  | Glyburide | 4264 |
| **Gastrointestinal** | | |
|  | Mineral oil | 4498 (only products for oral use), 42539, 43657, 46017 |
| **Analgesics** | | |
|  | Indomethacin | 4810 (only products for oral use) |
|  | Ketorolac | 46006, 47066 (only products for oral use) |
|  | Meperidine | 5603, 46412 |
|  | Pentazocine | 44528 |
| ***Muscle relaxants*** | Carisoprodol | 46167 |
|  | Chlorzoxazone | 46143, 46421 |
|  | Cyclobenzaprine | 38873, 46516 |
|  | Orphenadrine | 6019, 46098, 46183, 46871, 47570, 6734, 6747, 46094, 46254 |
| **Genitourinary** | | |
|  | Desmopressin | 38587, 46859 |

Table S2. Bayesian Information criteria for the latent class models, by number of groups, for two cohorts of individuals newly diagnosed with diabetes, according to sex, Quebec, Canada

| Models | People with a new diagnosis of diabetes | | People with a new diagnosis of diabetes and a comorbidity score of zero | |
| --- | --- | --- | --- | --- |
|  | Men subpopulation | Women subpopulation | Men subpopulation | Women subpopulation |
| 1 group | 133,943.1 | - | 45,213.0 | 47,185.1 |
| 2 groups | 122,852.4 | 118,329.5 | 42,105.7 | 43,631.2 |
| 3 groups | 118,017.7 | 113,385.8 | 40,436.6 | 41,784.9 |
| 4 groups | **115,451.7** | 110,946.4 | 39,731.8 | 40,968.4 |
| 5 groups | 115,452.2 | **109,785.0** | **39,629.2** | 40,445.1 |
| 6 groups | 115,452.2 | 109,785.9 | 39,262.7 | **40,333.2** |
| 7 groups | 115,452.2 | 109,785.9 | 39,262.6 | 40,235.7 |
| 8 groups | 115,452.2 | 109,785.9 | 39,262.9 | 40,235.4 |
| 9 groups | 115,452.2 | 109,784.7 | 39,262.9 | 40,235.4 |
| 10 groups | 115,452.2 | 109,785.6 | 39,262.9 | 40,235.4 |

***Legend.*** Bold values correspond to the number of selected groups based on the BIC.

1. Men
2. Women

Figure S1. Most frequently used Anatomical Therapeutic Chemical class of medications in individuals newly diagnosed with diabetes according to trajectory groups, by sex, in Quebec, Canada

***Legend.*** The numbers represent the percentage of medications classes used. For each trajectory group, the numerator refers to the number of individuals using a specific class of medications and the denominator to the number of individuals in that trajectory.

**A) Men, n = 5330**

**
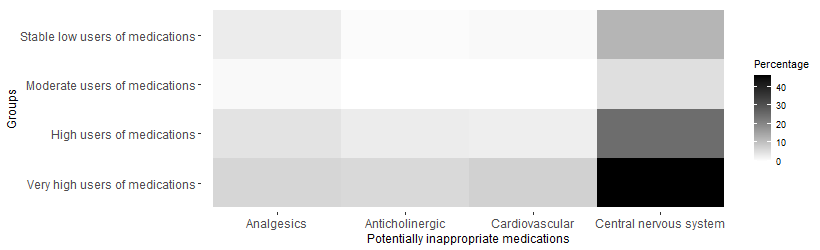
**

**B) Women, n = 5033**

**
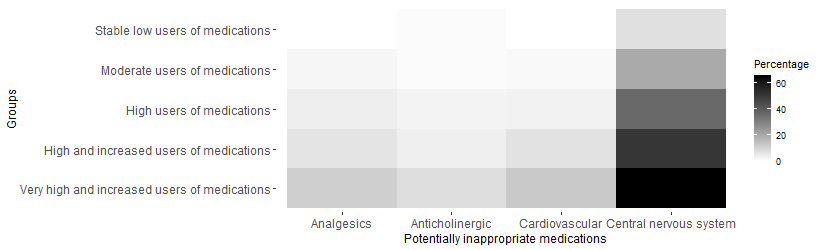
**

Figure S2. Use of potentially inappropriate medications in individuals newly diagnosed with diabetes according to trajectory groups, by sex, in Quebec, Canada

Table S3. Model adequacy criteria for the latent class models for medication use according to trajectory groups, by sex, Quebec, Canada

| Male |  | Stable low users of medications (n=766) | Moderate users of medications (n=1946) | High users of medications (n=1858) | Very high users of medications (n=760) |  |
| --- | --- | --- | --- | --- | --- | --- |
| Average posterior probability |  | 0.92 | 0.90 | 0.91 | 0.93 |  |
| Relative entropy | 0.85 |  |  |  |  |  |
| Odds of correct classification |  | 69.68 | 16.93 | 19.17 | 80.78 |  |
| Female |  | Stable low users of medications (n=552) | Moderate users of medications (n=1262) | High users of medications (n=1625) | High and increased users of medications (n=1205) | Very high users of medications (n=389) |
| Average posterior probability |  | 0.92 | 0.89 | 0.88 | 0.89 | 0.92 |
| Relative entropy | 0.84 |  |  |  |  |  |
| Odds of correct classification |  | 91.77 | 25.39 | 16.34 | 26.29 | 129.18 |

Table S4. Fit indices and model adequacy (100 bootstrap samples) in individuals newly diagnosed with diabetes, by sex, Quebec, Canada

|  | Men | Women |
| --- | --- | --- |
| Percentage of samples that identified | | |
| 4 groups | 59 | 7 |
| 5 groups | 4 | 86 |
| 6 groups | 35 | 7 |
| 7 groups | 2 | 0 |
| 8 groups | 0 | 0 |
| 9 groups | 0 | 0 |

**Supplementary material B. Sensitivity analysis**

**People newly diagnosed with diabetes with a comorbidity score of 0**

**Men subpopulation**

The BIC suggested six groups (Table S2), but because the relative entropy was less than 0.8, we considered a model with a number of groups of five. The five groups ranged from very low and increased users of medications to very high and increased users of medications (Figure S3, A). The two largest groups (“Moderate users of medications” and “High users of medications”) represented nearly two-thirds of the men subpopulation and had significant use of medications. “Very low and increased users of medications” consistently claimed a low number of medications with a small increase over time.

The five groups were significantly different according to age, number of medications and health services used were significantly different between the five groups (Table S5). The distribution of the classes of medications used was in the same order across the five groups, with the proportion of users increasing as one moves to a group with higher medication claims. Specifically, the classes of medications most frequently claimed in the five groups were lipid modifying agents, drugs used in diabetes, agents acting on the renin-angiotensin system and analgesics (Figure S4, A). Around 30% of “Very high and increased users of medications” had a claim for potentially inappropriate medications of the central nervous system (Figure S5).

**Women subpopulation**

Because the relative entropy of the optimal model suggested by the BIC was less than 0.8 (Table S2), we considered a model with six groups. The three largest groups (“Moderate users of medications”, “High users of medications” and “High and increased users of medications”) represented 73% of the women subpopulation and were characterized by an important use of medications (Figure S3, B). “Very low users of medications” (5.7%, 113/1977) represented the smallest group, as in the men subpopulation, and claimed on average less than 2 medications in 2015.

The six groups were different according to age, number of medications and health services used (Table S6). The distribution of the classes of medications used was not similar between the groups. The most frequent class of medications claimed in the two groups of high use of medications were lipid modifying therapies, drugs used in diabetes, agents acting on the renin-angiotensin system and analgesics. In the very high medication use group, drugs used in diabetes, lipid modifying therapies, analgesics, drugs for acid related disorders and agents acting on the renin-angiotensin system were the most frequently claimed (Figure S4, B). Approximately 50% of “Very high and increased users of medications” had a claim for potentially inappropriate medications of the central nervous system (Figure S5).

All three model adequacy criteria were above the threshold for men and women subpopulation respectively (Table S7).

**A) Men, n = 1962 B) Women, n = 1977**

**
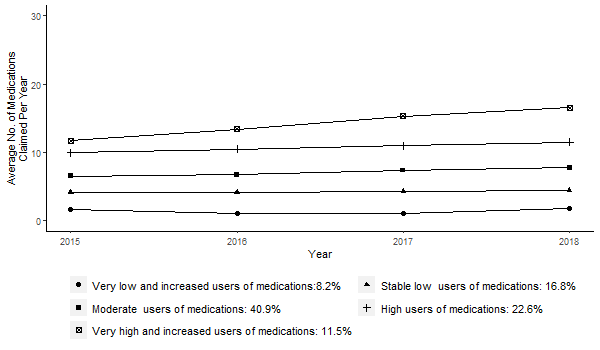

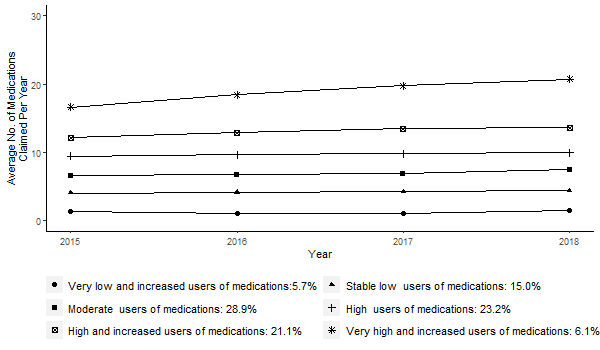
**

Figure S3. Trajectories of medications used among individuals with a new diagnosis of diabetes and a comorbidity score of zero, by sex, in Quebec, Canada, n=3939

Table S5. Characteristics of older men newly diagnosed with diabetes and with a comorbidity score of zero by trajectory groups, 2015, Quebec, Canada

|  | Very low and increased users of medications (n=161) | Stable low users of medications (n=329) | Moderate users of medications (n=803) | High users of medications (n=443) | Very high and increased users of medications (n=226) | p-value |
| --- | --- | --- | --- | --- | --- | --- |
| Age, mean (SD)^1^ | 71.2 (4.6) | 71.5 (4.1) | 72.2 (4.8) | 72.3 (4.6) | 72.7 (5.3) | 0.00661 |
| Residence area^2^, n (%) |  |  |  |  |  |  |
| Urban | 130 (80.7) | 257 (78.1) | 630 (78.5) | 324 (73.1) | 164 (72.6) | 0.0738 |
| Rural | 31 (19.3) | 72 (21.9) | 173 (21.5) | 119 (26.9) | 62 (27.4) |  |
| Material deprivation quintile^2^, n (%) |  |  |  |  |  |  |
| Quintiles 1 - 3 (privileged) | 86 (57.7) | 174 (55.4) | 400 (53.3) | 214 (51.1) | 117 (54.7) | 0.6229 |
| Quintiles 4 - 5 (deprived) | 63 (42.3) | 140 (44.6) | 350 (46.7) | 205 (48.9) | 97 (45.3) |  |
| Social deprivation quintile^2^, n (%) |  |  |  |  |  | 0.7145 |
| Quintiles 1 - 3 (privileged) | 90 (60.4) | 199 (63.4) | 474 (63.2) | 262 (62.5) | 144 (67.3) |  |
| Quintiles 4 - 5 (deprived) | 59 (39.6) | 115 (36.6) | 276 (36.8) | 157 (37.5) | 70 (32.7) |  |
| Number of medications used, mean (SD)^1^ | 1.6 (1.8) | 4.2 (1.3) | 6.5 (2.2) | 10.0 (2.4) | 11.7 (5.3) | <0.0001 |
| Number of visits to a generalist, mean (SD)^1^ | 1.6 (1.7) | 2.0 (1.7) | 2.6 (2.1) | 3.0 (2.2) | 3.6 (3.0) | <0.0001 |
| Number of visits to a specialist, mean (SD)^1^ | 1.5 (2.3) | 1.6 (2.5) | 2.5 (4.0) | 3.6 (4.2) | 4.8 (6.3) | <0.0001 |
| Number of emergency visits mean (SD)^1^ | 0.2 (0.5) | 0.1 (0.3) | 0.2 (0.6) | 0.3 (0.7) | 0.3 (0.7) | <0.0001 |
| Length (in days) of hospitalization, mean (SD)^1^ | 0.1 (0.5) | 0.1 (0.8) | 0.2 (1.5) | 0.5 (2.9) | 0.2 (1.2) | 0.0154 |

***Legend.***

**^1^**SD: Standard deviation

^2^Sum of counts for residence area, material and social deprivation quintiles does not equal the total number of subjects due to missing values.

Table S6. Characteristics of older women newly diagnosed with diabetes and with a comorbidity score of zero by trajectory groups, 2015, Quebec, Canada

|  | Very low users of medications (n=113) | Stable low users of medications (n=296) | Moderate users of medications (n=571) | High users of medications (n=459) | High and increased users of medications (n=418) | Very high and increased users of medications (n=120) | p-value |
| --- | --- | --- | --- | --- | --- | --- | --- |
| Age, mean (SD)^1^ | 71.8 (5.1) | 72.3 (5.2) | 72.7 (5.7) | 73.6 (5.6) | 73.9 (6.1) | 74.5 (5.8) | <0.0001 |
| Residence area^2^, n (%) |  |  |  |  |  |  | 0.0259 |
| Urban | 89 (78.8) | 245 (82.8) | 479 (83.9) | 350 (76.4) | 339 (81.3) | 90 (75.0) |  |
| Rural | 24 (21.2) | 51 (17.2) | 92 (16.1) | 108 (23.6) | 78 (18.7) | 30 (25.0) |  |
| Material deprivation quintile^2^, n (%) |  |  |  |  |  |  | 0.3427 |
| Quintiles 1 - 3 (privileged) | 55 (51.9) | 154 (54.2) | 288 (55.1) | 203 (48.4) | 187 (50.1) | 50 (48.5) |  |
| Quintiles 4 - 5 (deprived) | 51 (48.1) | 130 (45.8) | 235 (44.9) | 216 (51.5) | 186 (49.9) | 53 (51.5) |  |
| Social deprivation quintile^2^, n (%) |  |  |  |  |  |  | 0.3168 |
| Quintiles 1 - 3 (privileged) | 69 (65.1) | 158 (55.6) | 286 (54.7) | 233 (55.6) | 198 (53.1) | 52 (50.5) |  |
| Quintiles 4 - 5 (deprived) | 37 (34.9) | 126 (44.4) | 237 (45.3) | 186 (44.4) | 175 (46.9) | 51 (49.5) |  |
| Number of medications, mean (SD)^1^ | 1.3 (1.3) | 4.1 (1.5) | 6.6 (2.0) | 9.5 (2.0) | 12.1 (3.0) | 16.6 (5.2) | <0.0001 |
| Number of visits to a generalist, mean (SD)^1^ | 1.9 (2.1) | 2.1 (1.8) | 2.7 (2.0) | 3.1 (2.3) | 3.6 (2.6) | 4.7 (3.6) | <0.0001 |
| Number of visits to a specialist, mean (SD)^1^ | 1.6 (2.2) | 1.8 (2.4) | 2.2 (2.9) | 3.4 (4.5) | 3.8 (4.1) | 5.9 (6.4) | <0.0001 |
| Number of emergency visits, mean (SD)^1^ | 0.1 (0.6) | 0.1 (0.3) | 0.2 (0.5) | 0.2 (0.6) | 0.4 (0.8) | 0.6 (1.1) | <0.0001 |
| Length (in days) of hospitalization, mean (SD)^1^ | 0.1 (0.7) | 0 (0) | 0.1 (1.0) | 0.2 (1.7) | 0.4 (2.2) | 0.3 (1.4) | 0.0154 |

***Legend.***

**^1^**SD: Standard deviation

**^2^**Sum of counts for material and social deprivation quintiles does not equal the total number of subjects due to missing values.

**A) Men, n = 1962**

**B) Women, n = 1977**

Figure S4. Most frequently used ATC class of medications during the year of diabetes diagnosis in individuals with a comorbidity score of zero according to trajectory groups, by sex, in Quebec, Canada

***Legend.*** Empty cells (0) mean that the number of individuals who had a prescription for that medication class was less than five. To preserve confidentiality, the number cannot be reported when the number of individuals is less than five.

ATC: Anatomical Therapeutic Chemical

The numbers represent the percentage of medications classes used. For each trajectory group, the numerator refers to the number of individuals using a specific class of medications and the denominator to the number of individuals in that trajectory.

**A) Men, n = 1962
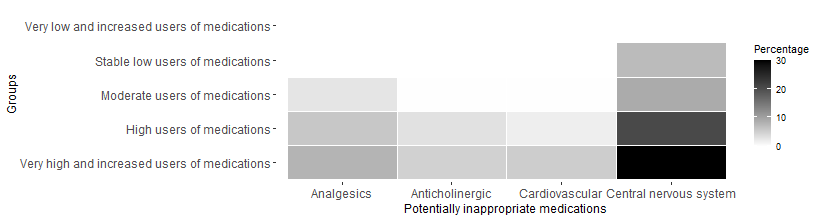
**

**B) Women, n = 1977**

**
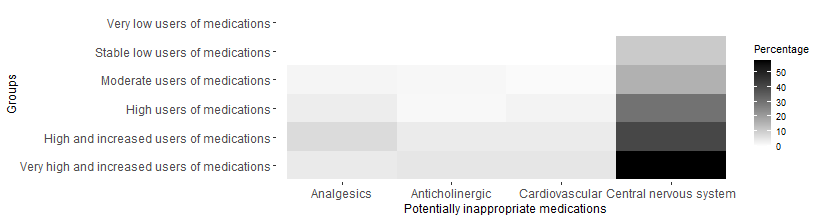
**

Figure S5. Use of potentially inappropriate medications during the year of diabetes diagnosis in individuals with a comorbidity score of zero according to trajectory groups, by sex, in Quebec, Canada

***Legend.*** Empty cells mean that the number of individuals who had a prescription for that medication class was less than five. To preserve confidentiality, the number cannot be reported when the number of individuals is less than five.

Table S7. Model adequacy criteria for the latent class models for medication use, people with a comorbidity score of zero according to trajectory groups, by sex, Quebec, Canada

| Men |  | Very low and increased users of medications (n=161) | Stable low users of medications (n=329) | Moderate users of medications (n=803) | High users of medications (n=443) | Very high and increased users of medications (n=226) |  |
| --- | --- | --- | --- | --- | --- | --- | --- |
| Average posterior probability |  | 0.9 | 0.9 | 0.9 | 0.8 | 0.9 |  |
| Relative entropy | 0.81 |  |  |  |  |  |  |
| Odds of correct classification |  | 160.1 | 36.3 | 11.0 | 17.0 | 65.2 |  |
| Women |  | Very low users of medications (n=113) | Stable low users of medications (n=296) | Moderate users of medications (n=571) | High users of medications (n=459) | High and increased users of medications (n=418) | Very high and increased users of medications (n=120) |
| Average posterior probability |  | 0.9 | 0.9 | 0.8 | 0.8 | 0.9 | 0.9 |
| Relative entropy | 0.80 |  |  |  |  |  |  |
| Odds of correct classification |  | 244.7 | 40.8 | 13.5 | 12.2 | 29.1 | 131.2 |
